# Simultaneous determination of 25 pesticides in *Zizania latifolia* by dispersive solid-phase extraction and liquid chromatography-tandem mass spectrometry

**DOI:** 10.1038/s41598-019-46523-y

**Published:** 2019-07-11

**Authors:** Feng Xu, Jia-yong Yu, Quan-sheng Wang, Yan Fu, Hao Zhang, Yin-liang Wu

**Affiliations:** 1 The Ningbo Academy of Agricultural Sciences, Ningbo, 315040 PR China; 2 SGS-CSTC Standards Technical Services (Ningbo) Co., Ltd., Ningbo, 315040 PR China

**Keywords:** Pollution remediation, Health occupations

## Abstract

An improved quick, easy, cheap, effective, rugged, and safe (QuEChERS) method combined with ultrapressure liquid chromatography tandem mass spectrometric method (UPLC-MS/MS) was developed to simultaneously determine 25 pesticides in *Zizania latifolia*. The samples were extracted with methanol(MeOH) and 0.1% formic acid (80:20, v/v) and cleaned with C_18_ absorbent and primary-secondary amine (PSA). LC separation was performed on a BEH C_18_ UPLC column under the condition of gradient elution with the mobile phase consisted of 0.5% formic acid (10 mM ammonium acetate)/MeOH. External standard calibration method with matrix-matched was used for quantification, and good linearity was obtained over a concentration range of 0.5–100 μg/l, with correlation coefficients greater than 0.9901. The limit of detection (LOD) and the limit of quantitation (LOQ) of the 25 pesticides were in the range of 0.2–1.0 µg/kg and 0.5–3.3 µg/kg, respectively. The recoveries ranged from 72% to 118%, and the relative standard deviations (RSDs) were less than 20%. Thus, the proposed method is suitable for the simultaneous determination of 25 pesticides in *Z. latifolia*.

## Introduction

*Zizania latifolia*, which is known as Manchurian wild rice, is the only member of the genus *Zizania* native to Asia^1^. The stems and grains of this plant used for food are edible. *Z. latifolia* is usually planted near rivers or the ocean because water is required over the entire period of growth. *Z. latifolia* is vulnerable to diseases and insects^2,3^. *Helminthosprium zizamae* Nishik and *Uromyces coronatus* Miyabeet Nishida frequently infest this plant and cause serious problems^4–6^. Five registered pesticides (Table 1) currently in use cannot control the diseases and pests of *Z. latifolia* in China due to pesticide resistance^7^. Thus, farmers frequently use unregistered pesticides on *Z. latifolia* to increase profits^1–3^. These unregistered pesticides mainly include triadimefon, prochloraz, carbendazim, isoprothiolane, tricyclazole, abamectin and nearly 14 other pesticides (Table 1)^1–3^. Consumer protection and the abuse of pesticides in agricultural production are of concern in China, and developing a rapid, effective and sensitive method to detect residues of pesticides in *Z. latifolia* is essential.

**Table 1:** LC -MS/MS parameters for 25 pesticides.

Analyte	Parent ion (m/z)	Product ion(m/z)	Dwell time(s)	Cone voltage(V)	Collision energy(eV)
Abamectin^a^	895.84	751.65, 183.20*	0.025	78	42,48
Acetamiprid	223.17	90.02, 125.91*	0.025	26	32,20
Buprofezin^a^	305.99	201.00, 105.96*	0.025	12	18,36
Carbendazim	191.97	132.10, 159.93*	0.025	24	28,18
Chlorantraniliprole	483.97	452.97, 285.98*	0.041	22	20,12
Difenoconazole	406.04	337.04, 250.96*	0.025	42	16,28
Diniconazole	326.14	172.97, 158.98*	0.025	34	34,34
Emamectin Benzoate^a^	886.84	126.08, 158.20*	0.025	48	38,34
Fenaminosulf	251.90	148.10, 172.11*	0.025	28	6,6
Flubendiamide	683.16	273.81, 407.86*	0.025	14	31,12
Hexaconazole	314.07	125.07, 159.06*	0.025	28	34,26
Imidacloprid	256.07	209.13, 175.06*	0.025	22	14,20
Iprodione	331.95	163.76, 246.88*	0.025	36	24,16
Isoprothiolane	291.10	189.01, 231.03*	0.025	16	20,10
Nitenpyram	271.07	196.04, 99.15*	0.025	20	18,16
Prochloraz^a^	376.22	265.94, 308.04*	0.025	20	16,10
Procymidone	284.07	255.98, 94.86*	0.025	60	16,22
Propiconazole^a^	341.64	123.21, 158.98*	0.025	34	50,28
Pymetrozine	218.03	78.65, 104.94*	0.025	30	30,18
Tebuconazole	308.19	165.04, 151.05*	0.025	28	30,36
Thiamethoxam	291.98	180.95, 210.99*	0.025	18	36,18
Thiophanate Methyl	343.03	310.94, 150.97*	0.041	20	12,20
Triadimefon	294.16	197.10, 69.06*	0.025	26	16,20
Triazophos	314.00	119.06, 162.00*	0.025	24	48,28
Tricyclazole	190.03	136.06, 163.07*	0.041	40	26,22

*Ion for quantification.

^a^Registered pesticide.

To analyse residual pesticides in biological samples, many methods such as immunoassay^8–12^, gas chromatography (GC)^13–15^, liquid chromatography (LC)^16–18^, gas chromatography-mass spectrometry (GC-MS)^19–21^, and liquid chromatography-mass spectrometry (LC-MS and LC-MS/MS)^22,23^, have been developed. Nevertheless, studies on *Z. latifolia* have mainly focused on its physiological and biochemical properties, and few reports have described methods for pesticide residue determination in this plant. Recently, Yang *et al*. established an LC-MS/MS method to determine emamectin benzoate and abamectin in *Z. latifolia*^24^, but so far, there are no available published data concerning analytical methods for more than 3 pesticide residues in *Z. latifolia*.

The quick, easy, cheap, effective, rugged, and safe (QuEChERS) method has been accepted worldwidely because of its adaptable, selective, simple and high-throughput analysis that does not require a mass of toxic organic solvents^25^. This method allows processing a significantly larger number of samples in a short amount of time. In the present study, an optimized QuEChERS method coupled with a excellent UPLC-MS/MS method was developed to simultaneously determine 25 pesticide residues in *Z. latifolia*.

## Materials and Methods

### Materials and reagents

Analytical standards of flubendiamide (97%), triadimefon (99%), tebuconazole (99%), difenoconazole (98%), carbendazim (98%), fenaminosulf (99%), and thiophanate-methyl (99%) were purchased from Dr. Ehrenstorfer GmbH (Augsburg, Germany), and analytical standards of triazophos, isoprothiolane, hexaconazole, propiconazole, prochloraz, tricyclazole, abamectin, buprofezin, emamectin benzoate, chlorantraniliprole, pymetrozine, imidacloprid, nitenpyram, thiamethoxam, acetamiprid, iprodione, procymidone, and diniconazole (all at 100 μg/ml) were bought from the Agro-Environmental Protection Institute (Tianjin, China). Methanol (MeOH; LC grade) and acetonitrile (ACN; LC grade) were obtained from Thermo Fisher Scientific, Inc. (Fairlawn, USA). Ammonium acetate (HPLC grade) and formic acid (HPLC grade) were provided by Tedia Company, Inc. (Fairfield, USA). Primary-secondary amine (PSA, 40–63 µm) and octadecyl silane (C_18_, 50 µm) sorbents were purchased from Shanghai Anpel Scientific Instrument Co., Ltd. (Shanghai, China). Purified water was prepared by a Milli-Q reagent water system (Millipore, Milford, MA, USA).

### Standard solutions

**Figure 1:**
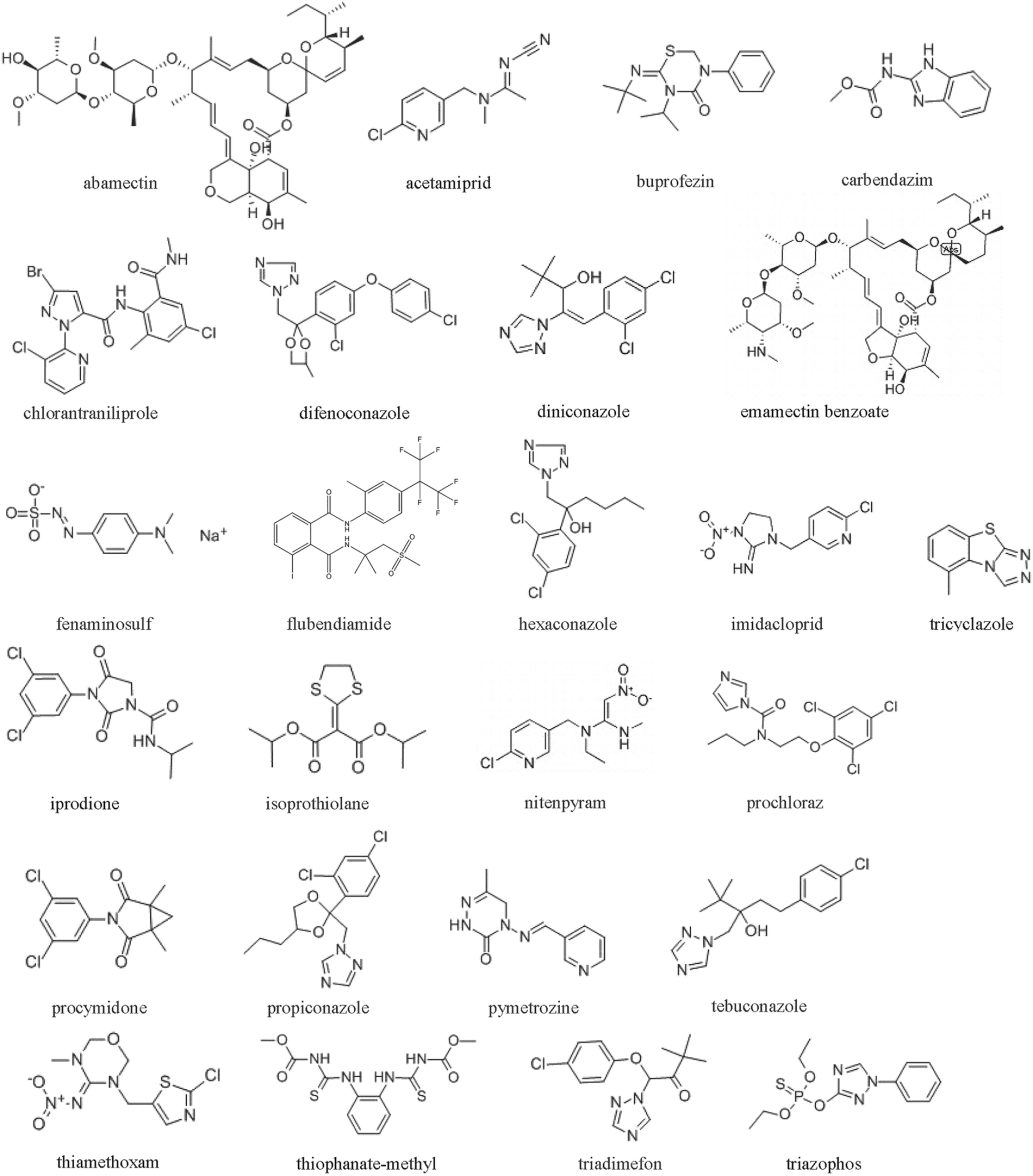
Chemical structures of the 25 pesticides.

Individual stock standard solutions of the 25 compounds (Fig. 1) at 100 μg/ml were prepared in MeOH. A mixed standard solution (4 μg/ml each) was prepared in MeOH by combining the 25 stock standard solutions and diluting with MeOH. Then, a 1.0 μg/ml mixed standard solution was made by diluting the 4 μg/ml mixed standard solution with MeOH and stored at −18 °C in the dark. Individual working solutions (1.0 μg/ml for each of the 25 compounds) for MS–MS optimization were prepared by diluting each stock solution with MeOH. Six mixed working standard solutions (2.5, 5.0, 10.0, 25.0, 50.0, and 100 μg/l) were established by diluting the 1.0 μg/ml mixed standard solution with 0.1% formic acid/MeOH (80:20, v/v).

### Chromatographic conditions

A Waters Acquity UPLC instrument (Milford, MA, USA) was used for analysis, and an Acquity BEH C_18_ column (2.1 mm × 100 mm, 1.7 μm) was utilized for separation while maintained at 35 °C. The mobile phase consisted of solvent A (0.5% formic acid containing 10 mM ammonium acetate) and solvent B (MeOH). The initial gradient conditions were set at 20% B and held for 1.1 min. Then, the gradient was increased linearly to 90% B at 3.5 min and maintained for 4.5 min. then the gradient was programmed to return to the initial conditions at 8.1 min to re-equilibrate the column for 1.9 min. The flow rate was 0.30 ml/min. Total run time of one injected sample was 10 minutes with the injection volume of10 µl in full-loop injection mode.

### Mass spectrometry conditions

MS/MS detection was performed on a Waters Xevo TQ triple-quadrupole MS system equipped with an electrospray ionization (ESI) source operated in positive mode. The ion source and desolvation temperatures were optimized at 150 °C and 500 °C, respectively. The capillary voltage and the flow rate of the desolvation gas (N_2_) were set at 2.2 kV and 1000 L/h, respectively. The collision cell pressure was 3.0 mbar sustained by the collision gas argon. Detection was carried out in a multiple-reaction monitoring (MRM) mode. Other parameters are shown in Table 1.

### Sample preparation

A homogeneous sample (5 g) was weighed, placed in a 50 ml polypropylene centrifuge tube, and 10 ml of 0.1% formic acid/MeOH (20:80, v/v) was added to sample. The mixture was homogenized for 1 min using a high-speed dispersing device (Ultra-Turrax T 25; IKA, Germany) and vortexed for 1 min. The tube was subsequently centrifuged at 9500 rpm for 5 min, and a 1 ml aliquot was transferred to a tube containing PSA solid-phase extraction (SPE) sorbent (75 mg) and ODS C_18_ sorbent (75 mg). Next, the tube was vortexed for 30 s and centrifuged at 9500 rpm for 1 min. An aliquot of the supernatant (0.2 ml) was transferred to a new glass tube, reconstituted in 0.8 ml of 0.1% formic acid/MeOH (95:5, v/v), and vortexed for 15 s. The sample supernatant was subsequently passed through a 0.22 µm filter (Jinteng, Tianjin, China).

### Matrix effects

To evaluate the matrix effects, six concentrations (2.5, 5.0, 10.0, 25.0, 50.0, and 100 μg/l) of the 25 pesticides in pure solvent and a blank sample were analysed. The slope ratio of 25 pesticides was obtained by calculating the quotient of the matrix-matched calibration slope and the solvent calibration slope.

### Method validation

Analytical performance was examined in terms of the selectivity, linearity, mean recovery, repeatability, LOD and LOQ of the method in accordance with the SANCO document^26^.

To confirm the absence of interfering substances around the retention times of the 25 pesticides, 20 blank samples were analysed.

Linearity was evaluated using matrix-matched standard solutions prepared as described in section 2.5 at six concentrations between 0.5 and 100 µg/l (2.5, 5.0, 10.0, 25.0, 50.0, and 100 µg/l for fenaminosulf, procymidone and hexaconazole; 0.5, 1.0, 5.0, 10.0, 50.0, and 100 µg/l for the other compounds). Excellent linearity was based on a high coefficient of determination (*r*^2^).

The recoveries and repeatability (intra-day and inter-day) of the method were determined with spiked blank samples at three concentrations (0.05, 0.1 and 0.25 mg/kg for fenaminosulf, procymidone, and hexaconazole; 0.01, 0.05 and 0.1 mg/kg for the other compounds). The intra-day repeatability was determined with five replicates at each calibration level on the same day, and the inter-day repeatability was calculated from five replicates at 0.05 mg/kg per day over 3 consecutive days. The intra-day and inter-day repeatability values were expressed as the relative standard deviation (RSD).

The LOD and LOQ were calculated from the signal-to-noise ratio (S/N) of a chromatographic peak, where LOD = 3 S/N and LOQ = 10 S/N.

## Results and Discussion

### LC-MS/MS optimization

In this study, positive mode produced higher precursor ion signal intensities than the negative mode for all pesticides. Therefore, the analysis of target compounds.is carried out in the [M + H]^+^ ion mode as the precursor ion. One parent ion and two transitions were chosen. The most intense transition was used for quantitation^27^, while the other transition was employed for qualitative. The optimal parameters for each compound are shown in Table 1.

**Figure 2:**
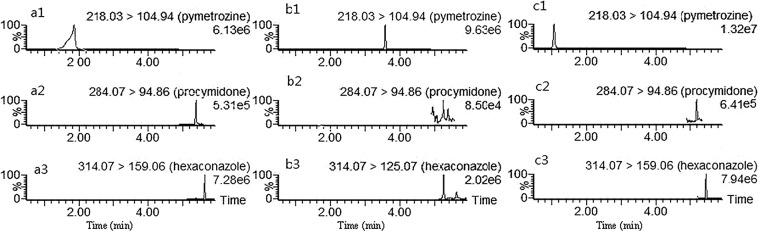
MRM chromatograms of matrix standard solutions at 10 μg/l generated with 0.1% formic acid/MeOH as the mobile phase (**a**), 0.1% formic acid (10 mM ammonium acetate)/MeOH as the mobile phase (**b**), or 0.5% formic acid (10 mM ammonium acetate)/MeOH as the mobile phase (**c**).

After optimizing the MS cinditions, the mobile phase composition was explored by the chromatographic column. It is well known that the [M + H]^+^ ion forms easily under acidic conditions. Therefore, 0.1% formic acid/ACN and 0.1% formic acid/MeOH solutions were first investigated. Satisfactory separation was difficult to achieve for the 9 triazole pesticides when 0.1% formic acid/ACN solution was used, while the peak shape of pymetrozine was poor when 0.1% formic acid/MeOH was used (Fig. 2a). To achieve both these goals simultaneously, the 0.1% formic acid solution was replaced with 0.1% formic acid containing 10 mM ammonium acetate. With this solvent system, the separation of the 9 triazole pesticides did not improve significantly, but the peak shape of pymetrozine improved (Fig. 2b). Thus, 0.1% formic acid (10 mM ammonium acetate)/MeOH was chosen initially. However, the ionization of procymidone and hexaconazole was suppressed in this mobile phase (Fig. 2b). The responses of procymidone and hexaconazole obviously increased when the concentration of formic acid was changed from 0.1% to 0.5% (Fig. 2c). Thus, 0.5% formic acid (10 mM ammonium acetate)/MeOH was finally chosen as the mobile phase in the current study. Moreover, in order to improve the sensitivity of all compounds, the chromatogram was divided into five regions.

### Optimization of sample preparation

Salting-out assisted water–acetonitrile extraction is an convenient sample preparation technique when pesticide residue analytical method development. Compared with traditional liquid-liquid extraction and SPE, this method is more environmentally friendly, more cost-efficient and faster. Hence over the past decade, it has obtained growing interest in QuEChERS sample preparation^28–30^. However, salting out was not used in the present study because fenaminosulf and pymetrozine are highly soluble in water. To achieve satisfactory recoveries for all target compounds from the *Z. latifolia* samples, three extraction solvents (0.1% formic acid/ACN (20:80, v/v), 0.1% formic acid/MeOH (20:80, v/v) and ACN) were evaluated at a fortification level of 50 µg/kg. The best recoveries for most of the compounds were obtained with 0.1% formic acid/MeOH (20:80, v/v), which was selected as the optimal extraction solvent.

**Figure 3:**
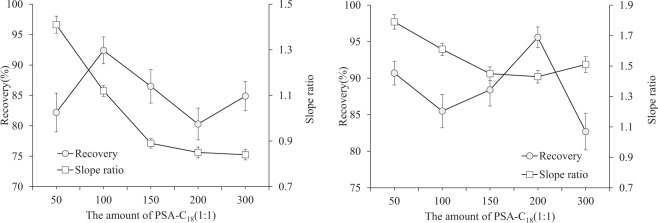
Effect of different amounts of PSA-C_18_ (1:1) on the recovery of fenaminosulf (**a**) and iprodione (**b**) and ratios of the external calibration slopes for the matrix-matched standards to the external calibration slopes for the standards in solvent. (Mean ± SD).

*Z. latifolia* mainly contains carbohydrates, proteins and fats. Pesticides with high polarity are highly susceptible to interferences from impurities. To reduce the level of the co-extracted matrix, and obtain good purification efficiency, a simple and effective clean-up procedure with dispersive SPE (dSPE) is often used. The original QuEChERS method involves cleaning up with PSA sorbent^31^. PSA can effectively adsorb organic acids, fatty acids, sugar, and other interferences in the matrix. However, compounds with carboxyl groups are easily retained by PSA. The QuEChERS method has been modified to enable the use of C_18_ for clean-up, and strong adsorption of low-polarity matrix interferences such as fatty acids, olefins, and large molecules, such as sterols and pigments has been achieved^32,33^. In the present study, a mixed PSA-C_18_ (1:1) sorbent was used for clean-up. The effects of the amount of PSA-C_18_ (1:1) sorbent (50–300 mg) on the matrix effect and recoveries were examined in detail (Fig. 3). The matrix effect was counted by the following formula: matrix effect = (external calibration slope for matrix-matched standards/external calibration slope standard in solvent)^34,35^. For most compounds, the recoveries were above 90%, and the matrix effect did not obviously change when the amount of PSA-C_18_ (1:1) was varied from 50 to 300 mg. However, there were significant differences in the recoveries and matrix effects of fenaminosulf and iprodione when the amount of PSA-C_18_ (1:1) was increased from 50 to 300 mg (Fig. 3). According to the data in Fig. 3, 150 mg of PSA-C_18_ was selected as the optimal sorbent amount.

### Matrix effect

The detector response of pesticides may be influenced by co-extracted materials from the sample. To evaluate the matrix effect, the ratio of the external calibration slope for the matrix-matched standard and the external calibration slope for the standard in solvent was compared for each target compound (Table 2). According to the study of Frenich *et al*., when the value is between 0.8 and 1.2, signal suppression or enhancement by matrix components can be tolerated^36^. The values of four compounds fell outside this range and indicated signal enhancement by the matrix effect. Therefore, matrix-matched standard solutions were used for quantification in this study.

**Table 2:** Mean recoveries, RSDs, the linearity, regression coefficients of standard curves (*r*^2^), matrix effects, LOD and LOQ (μg/kg) of 25 pesticides in *Zizania latifolia* by LC-MS/MS.

Analyte	*Zizania latifolia* ^a^			Inter-day RSDs	Linear equation	*r* ^2^	Matrix effects	LOD	LOQ
	0.01 mg/kgb	0.05 mg/kg	0.10 mg/kg						
Abamectin	89 (7)	87 (5)	83 (6)	9	*y* = 1.1 × 10^2^*x*-1.5 × 10^2^	0.9999	1.5	0.2	0.5
Acetamiprid	117 (12)	115 (10)	109 (14)	15	*y* = 1.2 × 10^4^*x* + 1.0 × 10^5^	0.9966	1.1	0.2	0.6
Buprofezin	91 (15)	95 (16)	87 (6)	18	*y* = 3.9 × 10^4^*x* + 5.4 × 10^5^	0.9903	1.1	0.3	1.0
Carbendazim	81 (5)	82 (2)	87 (4)	6	*y* = 1.8 × 10^4^*x* + 4.3 × 10^5^	0.9980	0.7	0.3	1.0
Chlorantraniliprole	118 (3)	90 (2)	110 (6)	7	*y* = 3.3 × 10^3^*x* + 1.1 × 10^4^	0.9993	1.1	0.4	1.1
Difenoconazole	72 (8)	107 (11)	90 (13)	14	*y* = 7.4 × 10^3^*x* + 2.2 × 10^3^	1.0000	1.1	0.2	0.7
Diniconazole	90 (3)	107 (7)	95 (8)	10	*y* = 1.4 × 10^3^*x* + 8.7 × 10^3^	0.9975	1.1	0.3	0.6
Emamectin Benzoate	76 (8)	75 (5)	80 (8)	10	*y* = 4.3 × 10^4^*x* + 2.3 × 10^5^	0.9981	1.1	0.2	0.7
Fenaminosulf	72 (11)	75 (12)	74 (8)	14	*y* = 1.2 × 10^2^*x* + 1.3 × 10^3^	0.9970	0.6	1.0	3.3
Flubendiamide	88 (7)	80 (4)	72 (9)	12	*y* = 2.3 × 10^2^*x* + 3.0 × 10^2^	0.9954	1.3	0.2	0.7
Hexaconazole	89 (14)	88 (8)	77 (6)	18	*y* = 2.9 × 10^3^*x* + 1.9 × 10^4^	0.9978	1.0	0.8	2.5
Imidacloprid	91 (9)	96 (8)	91 (10)	16	*y* = 2.7 × 10^3^*x* + 1.4 × 10^4^	0.9990	1.1	0.5	1.5
Iprodione	88 (4)	87 (7)	80 (4)	12	*y* = 1.4 × 10^3^*x* + 1.7 × 10^4^	0.9910	0.9	0.3	0.8
Isoprothiolane	117 (4)	93 (6)	91 (10)	19	*y* = 2.3 × 10^4^*x* + 3.5 × 10^5^	0.9938	1.0	0.3	0.9
Nitenpyram	85 (15)	87 (8)	89 (10)	16	*y* = 1.1 × 10^4^*x* + 1.2 × 10^5^	0.9937	0.8	0.3	1.1
Prochloraz	105 (8)	100(5)	107 (6)	9	*y* = 4.2 × 10^4^*x* + 1.2 × 10^5^	0.9994	1.1	0.2	0.6
Procymidone	82 (2)	81 (6)	90 (5)	8	*y* = 3.5 × 10^2^*x*-22	0.9984	1.1	0.8	2.5
Propiconazole	81 (2)	73 (5)	95 (9)	11	*y* = 1.1 × 10^4^*x* + 7.0 × 10^4^	0.9974	1.0	0.2	0.7
Pymetrozine	87(2)	85 (2)	90 (7)	8	*y* = 2.6 × 10^4^*x*-2.3 × 10^5^	0.9955	0.9	0.4	1.2
Tebuconazole	95 (7)	96 (6)	85 (5)	13	*y* = 7.5 × 10^2^*x* + 1.6 × 10^3^	0.9997	1.1	0.2	0.7
Thiamethoxam	80 (4)	80 (2)	91 (6)	8	*y* = 2.5 × 10^3^*x* + 2.1 × 10^4^	0.9966	1.0	0.3	1.0
Thiophanate methyl	99 (2)	91 (8)	93 (8)	10	*y* = 1.6 × 10^4^*x* + 9.9 × 10^4^	0.9969	1.2	0.3	1.0
Triadimefon	115 (11)	79 (12)	75 (10)	17	*y* = 3.5 × 10^4^*x* + 2.7 × 10^4^	1.0000	1.1	0.2	0.6
Triazophos	110 (3)	75 (8)	72 (8)	11	*y* = 2.9 × 10^4^*x* + 3.5 × 10^5^	0.9942	1.1	0.2	0.7
Tricyclazole	114 (5)	77 (4)	97 (7)	13	*y* = 1.2 × 10^4^*x* + 1.3 × 10^5^	0.9958	1.0	0.4	1.3

^a^Repeatability values, expressed as RSD, are given in brackets (n = 5).

^b^0.05, 0.1 and 0.25 mg/kg for fenaminosulf, procymidone and hexaconazole.

### Method validation

#### Selectivity

**Figure 4:**
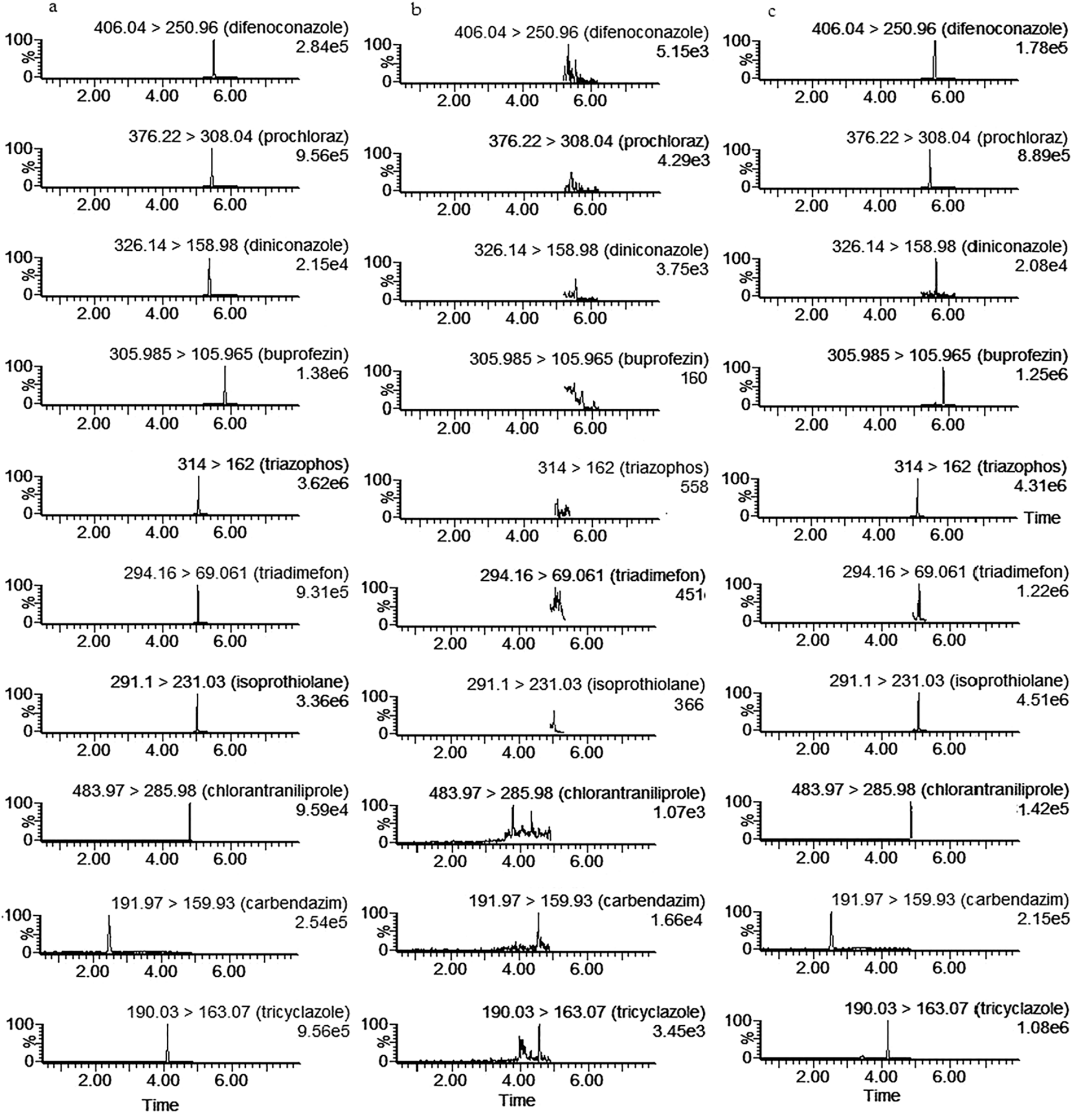
Representative MRM chromatograms of a working standard solution (1.0 μg/l for the 25 pesticides except fenaminosulf, procymidone and hexaconazole) (**a**), a blank sample (**b**) and a spiked blank sample (0.01 mg/kg for the 25 pesticides except fenaminosulf, procymidone and hexaconazole) (**c**).

To evaluate the selectivity, 20 blank samples of *Z. latifolia* were analysed. At the retention times of the 25 compounds, there were no interfering peaks were observed (Fig. 4b). Therefore, the selectivity of the analysis was sufficient.

#### Linearity

A matrix-matched calibration curve was established by determining the peak area of each pesticide standard over a concentration range of 0.5–100 µg/l (Table 2). The calibration curves of all of the pesticides had excellent linearity, with correlation coefficients (*r*^2^) between 0.9901 and 1.000.

#### Recovery and precision

Recoveries were determined by spiking three different concentrations of the pesticides into the blank samples. Next, all of the samples were extracted and analysed following the procedure described previously. The results are shown in Table 2. Single-point calibration with the matrix-matched standard solutions (1.0, 5.0, 10.0, and 25 μg/l for a 0.01, 0.05, 0.1, and 0.25 mg/kg fortified level, respectively) was conducted in the recovery test. The MRM chromatogram of a matrix-matched standard solution (1.0 μg/l) is shown in Fig. 4a. The mean recoveries varied from 72% to 118%, intra-day RSDs varied from 2% to 16%, and inter-day RSDs varied from 6% to 19%, respectively (Table 2). The good recoveries (70%–120%) and RSDs (≤20%) were in compliance with the requirements of the SANCO document^26^. These results demonstrated that the proposed method could achieve satisfactory recovery and precision for residue analysis in *Z. latifolia*. Representative chromatograms of the 25 pesticides in the blank and spiked samples are shown in Fig. 4b,c.

#### LOD and LOQ

As listed in Table 2, the ranges of the LODs and LOQs, calculated at S/N ratio = 3 and S/N ratio = 10, were 0.2–1.0 µg/kg and 0.5–3.3 µg/kg, respectively, for all of the compounds in the *Z. latifolia* matrix.

#### Real sample analysis

*Z. latifolia* is a vegetable consumed daily and is associated with severe pesticide abuse. In the final phase of this work, the validated QuEChERS method was utilized to measure the pesticide levels in 20 samples purchased from various markets in Ningbo (Zhejiang Province, China). Procymidone was detected at concentrations ranging from 0.005 mg/kg to 0.008 mg/kg in 3 samples, which are below the MRLs established by the EU, that is, 0.01 mg/kg for root and tuber vegetables. No other pesticides were detected in these samples.

## Conclusion

A rapid method was developed to analyse multiclass pesticide residues in *Z. latifolia* samples through UPLC-MS/MS with a QuEChERS method. Good recovries obtained via spiking blank samples infered that this method was enough reliable to analyze. The LODs and LOQs were sufficiently low to monitor the residues of 25 pesticides in the samples. This fast and convenient method was used for 20 actual real samples analysis, procymidone was detected in 3 samples.
